# Assessment of patients referred to urgent start peritoneal dialysis: when does the nurse contraindicate?

**DOI:** 10.1590/2175-8239-JBN-2020-0072

**Published:** 2020-10-12

**Authors:** Helen Caroline Ferreira, Fabiana Baggio Nerbass, Viviane Calice-Silva

**Affiliations:** 1 Centro de Tratamento de Doenças Renais de Joinville, Joinville, SC, Brasil.; 2 Fundação Pró-Rim, Joinville, SC, Brasil.

**Keywords:** Peritoneal Dialysis, Renal Insufficiency, Chronic, Renal Replacement Therapy, Diálise Peritoneal, Insuficiência Renal Crônica, Terapia de Substituição Renal

## Abstract

**Introduction:**

Urgent-start peritoneal dialysis (US-PD) has been used worldwide with very positive results. The prior assessment of candidates for this therapy by a nurse can favor the success of the therapy.

**Objectives:**

To identify the profile of patients who are candidates for US-PD, the causes of contraindication of the method by the nurse and their permanence in the method after 30 days, as well as the growth of the service after implementing the program.

**Methods:**

We retrospectively analyzed the forms used to assess candidates for US-PD applied by nurses between May 2017 and August 2019 in a clinic in Santa Catarina. We analyzed information on demographic profile, reasons for contraindication and permanence in the method after 30 days, as well as service growth after the program was implemented.

**Results:**

Of the 215 patients indicated for US-PD, 51% were male, 55% were under 60 years old, 51% had diabetes mellitus and 89% were hypertensive. Of these, 173 (80%) patients had the nurse’s approval for PD. The only cause contraindicated was the inability to self-care associated with the lack of family support. In the first 30 days after the assessment, 89% of the patients who started PD remained on it. During the study period, the PD service grew by 91%.

**Conclusion:**

During the study period, a fifth of patients referred to US-PD received contraindication by nursing due to self-care inability associated with the lack of family support. After 30 days, 89% of the patients remained on it.

## Resumo

**Introdução:**

A diálise peritoneal (DP) de início urgente ou *urgent start* (DP-US) vem sendo utilizada mundialmente e apresenta resultados bastante positivos. A avaliação prévia dos candidatos a essa terapia por um enfermeiro pode favorecer o sucesso do tratamento.

**Objetivos:**

Identificar o perfil dos pacientes candidatos à DP-US, as causas de contraindicação do método pelo enfermeiro e sua permanência após 30 dias, bem como o crescimento do serviço com a implementação do programa.

**Métodos:**

Foram analisados retrospectivamente formulários de avaliação dos candidatos à DP-US aplicados pelos enfermeiros entre maio de 2017 a agosto de 2019 em uma clínica de diálise localizada na região sul brasileira. Foram analisadas informações referentes a perfil demográfico, motivos para contraindicação e permanência no método após 30 dias, bem como crescimento do serviço após implementação do programa.

**Resultados:**

Dos 215 pacientes indicados para DP-US, 51% eram do sexo masculino, 55% tinham menos de 60 anos, 51% apresentavam diabetes *mellitus*, 89% eram hipertensos. Desses, 173 (80%) pacientes obtiveram parecer positivo do enfermeiro para DP. A única causa para contraindicação foi a incapacidade para autocuidado associado à falta de apoio familiar. Nos primeiros 30 dias após a avaliação, 89% dos pacientes que iniciaram a DP-US permaneceram na técnica. No período do estudo, o serviço de DP teve um crescimento de 91%.

**Conclusão:**

Um quinto dos pacientes indicados à DP-US recebeu contraindicação pela enfermagem devido à incapacidade de autocuidado associado à falta de apoio familiar. Após 30 dias, 89% dos pacientes haviam permanecido na técnica.

## Introduction

Chronic kidney disease (CKD) is a major public health problem worldwide. When progressing to the final stage, it is necessary to initiate renal replacement therapies (RRT) for the maintenance of life. In Brazil, in 2019, there were more than 139 thousand patients undergoing dialysis, and approximately 93.2%, on hemodialysis (HD).^1^

The late referral to RRT of these patients with previously unknown CKD and unpredictable deterioration of renal function remains a major problem in our country, leading to the need for unplanned dialysis initiation. Due to the lack of adequate vascular access in these situations, these patients are usually submitted to HD through a central venous catheter (CVC), which is directly associated with a greater risk of infections and hospitalizations, increasing mortality rates, especially in the first 90 days.^2^

As an alternative, there has been a significant increase in clinical experience with unplanned onset PD in the past decade, which, in turn, has shown excellent results. Unplanned PD, also known as urgent start PD (US-PD), has several definitions in the literature, ranging from when PD starts in the first 72 hours to 14 days after implant. Peritoneal dialysis (PD) has been considered a viable and safe alternative for urgent dialysis initiation in both developed and developing countries, making it also a useful tool for increasing the number of patients in this therapy in various services.^3^

This modality offers the advantage of preserving vascular access and is also associated with better preservation of patients' residual renal function (RRF). This scenario can change the reality of many dialysis centers that are already overcrowded, with no vacancies for HD in addition to offering some additional benefits to patients, such as greater flexibility in therapy and reduction of dietary restrictions, when compared to HD.^4^^,^ 5

Unlike HD, which is usually performed in dialysis clinics about three times a week in four-hour sessions, PD treatment is a home therapy with the patient himself or his caregiver (s) being responsible (s) by therapy. The initial contact of the nurse with the patient and the caregivers in the approach to therapy make it possible to identify difficulties associated with self-care or family support, factors related to the permanence of these patients in therapy and its long-term success.^6^

This study aimed to identify the profile of patients candidates for US-PD evaluated in the service between May 2017 and August 2019, the causes of nurses' contraindication and the permanence in the technique after 30 days, as well as the growth of the service after implementation of the US-PD program.

## Methodology

### Study Design

The forms used by the nurses at the service were retrospectively analyzed concerning the evaluation of patients referred to the US-PD after the medical team's opinion between May 2017 and August 2019.

### Study Site

The study was carried out in a dialysis clinic northern part of Santa Catarina state.

### Patients

The patients were referred for evaluation with the nursing team after consultation with a nephrologist, either on an outpatient basis or while hospitalized, with an indication for urgent dialysis initiation. All referred patients did not have definitive access to RRT and had no clinical contraindications for PD. All patients who started treatment were evaluated by the nurse and no patient contraindicated by that professional started therapy.

### Nursing Evaluation

Nurses used a standard service form for the evaluation, which contained questions that addressed:

- Demographic characteristics: origin, race, sex and age.
- Clinical info: comorbidities, symptoms, surgeries.
- Perception of the nurse for self-care and family support.

**Figure 1:**
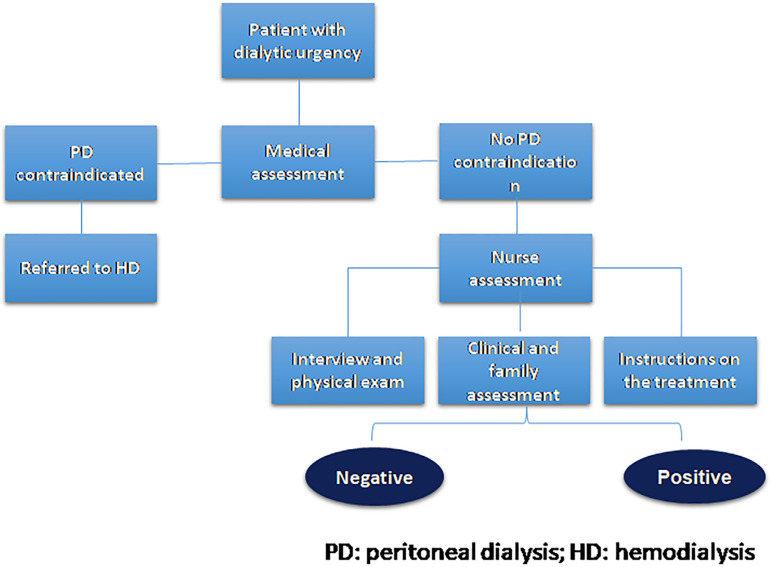
Patient evaluation flowchart.

Based on this information, the nurse issued the opinion, indicating PD therapy or not. The criteria for indicating were based mainly on the perception of self-care and family support, analyzing the interest of those involved in the therapy, associated with the patient's autonomy or the existence of a caregiver to assist the patient. With a positive opinion, the patient was referred to the implantation of the peritoneal catheter and initiation of dialysis. The permanence or not of these patients on PD was verified after 30 days of the first contact, a period considered critical for leaving the technique due to the adaptation and the clinical demands of the patient (Figure 1).

## Results

Table 1 shows the main characteristics of the studied group. There was no gender predominance, 55% of the patients were adults under 60 years of age. Half had diabetes mellitus (51%), and the vast majority (89%) had arterial hypertension as comorbidity. The main underlying disease diagnosed was hypertensive nephrosclerosis (Table 1).

**Table 1:** Characteristics of the study population (n = 215)

Age (years)	56.6 ± 14.1
Male n (%)	109 (51)
Caucasian n (%)	176 (82)
Diabetes *mellitus* n (%)	109 (51)
Hypertension (%)	191 (89)
CKD etiology	
Hypertensive nephrosclerosis n (%)	85 (39)
Diabetic nephropathy n (%)	78 (36)
Glomerulopathies n (%)	21 (10)
Polycystic kidney disease n (%)	4 (2)
Undetermined n (%)	17 (8)
Others n (%)	10 (5)

Of the 215 patients evaluated, 173 (80%) obtained a positive evaluation for home therapy 42 (20%) were contraindicated by the nurse. In all cases, the contraindication was the observation of the incapacity for self-care associated with the lack of a caregiver.

**Graph 1:**
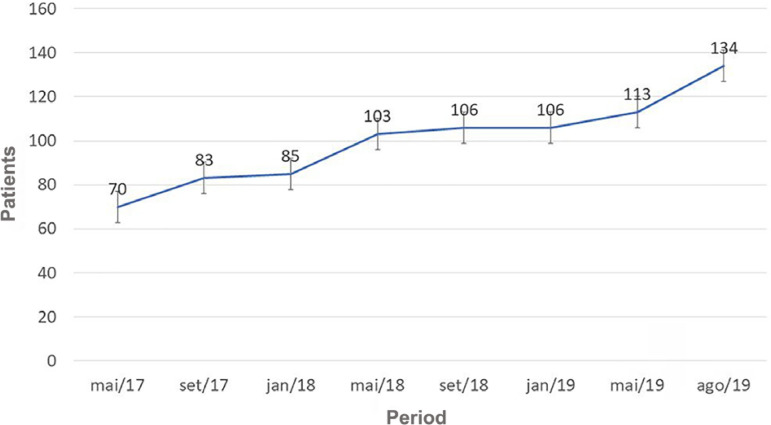
Number of patients on peritoneal dialysis after starting US_PD.

After 30 days of the nurses' evaluation, we found that 37 (21%) patients of the 173 who obtained a positive opinion recovered renal function and no longer needed RRT in the period. Thus, 136 patients actually started on PD. Of these, 15 (11%) chose to migrate to HD in the period. Of the 121 (89%) who remained on the technique, 96% chose automated peritoneal dialysis (APD) and only 1 (4%) chose continuous ambulatory peritoneal dialysis (CAPD). After the implementation of US-PD, the program grew by 91% in 28 months (Graph 1).

## Discussion

The present study showed that 20% of the patients referred to US-PD were contraindicated by the nursing team. All of them due to lack of self-care capacity or caregiver for assistance. These factors must be considered when defining PD as a treatment. Studies show that family support is a primary factor for the choice and success of home therapy for patients with incapacity for self-care.^7^^,^ 8 After 30 days, 89% who started on PD remained on the technique.

The choice of PD should be a joint decision of the patient with family and the dialysis team. In order to carry out the treatment at home, it is necessary that family members and, if possible, the patient undergo training provided by nurses in this technique. The bond and family support are of fundamental importance for the good progress of the therapy success and the patient care.^8^

It is a great challenge for dialysis clinics to maintain the growth of PD services and the maintenance of these patients in the treatment. With the ageing of the population and the improvement in survival, patients with CKD reach the most advanced stages of the disease, getting older, when they are more debilitated, dependent and with difficulties for self-care, factors that hinder the implementation of home therapy in this group of patients. In addition, even when younger, the multiple comorbidities and consequences, such as visual loss, in the case of diabetic patients, amputations, among others, may end up impairing the accomplishment of home therapy without assistance. For these patients, support from family members is crucial. When this support is not available, treatment ends up being contraindicated.^8^^,^ 9

Recent studies on PD incident patients in Brazil (BRAZPD) showed a sociodemographic and clinical profile similar to those identified in this analysis, our study had a higher proportion of Caucasian patients (BRAZPD 60%), due to predominant European colonization in the study region. SAH and DM were the most prevalent comorbidities, corroborating with national data, in which 6,198 patients were evaluated; of these, 40% had diabetes mellitus and 90% had hypertension.^9^ As for the choice of the modality, 96% of the patients chose the automated modality, which confirms the national preference for automated peritoneal dialysis (APD-72.5%).^10^

The US-PD program provided the institution with a 91% growth in the total number of PD patients in 28 months. In another Brazilian sudy, the impact on the growth of the PD program was 41% in 6 months.^11^

Our study has some limitations inherent to its retrospective design. However, as a strong point we emphasize that this is one of the first studies that address the role of nurses in the selection of patients for PD, especially in the scenario of urgent dialysis start. Despite the lack of objective tools for the assessment of patients and family members, the nurse's previous contact improves the understanding of the support needed to perform the therapy, increasing the likelihood of compliance and success treatment in the long term.

## Conclusion

This study showed that the profile of candidates for US-PD is similar to the PD population in our country. A fifth of the candidates for US-PD were contraindicated by the nurse, all due to incapacity for self-care associated with the lack of a caregiver. Regarding the assessed outcomes, after 30 days on PD, 89% of the patients remained on the technique; after the implementation of the US-PD program, the PD service grew by 91% in the number of patients.
